# Identifying collateral and synthetic lethal vulnerabilities within the DNA-damage response

**DOI:** 10.1186/s12859-021-04168-7

**Published:** 2021-05-15

**Authors:** Pietro Pinoli, Sriganesh Srihari, Limsoon Wong, Stefano Ceri

**Affiliations:** 1 Department of Electronic, Information and Bioengineering, Politecnico di Milano, Piazza Leonardo da Vinci 32, Milan, Italy; 2 School of Computing, National University of Singapore, Computing Drive 13, Singapore, Singapore; 3 Institute for Molecular Bioscience, The University of Queensland, St Lucia, QLD, Australia

**Keywords:** Synthetic lethality, Copy number alteration, DNA damage repair genes

## Abstract

**Background:**

A pair of genes is defined as synthetically lethal if defects on both cause the death of the cell but a defect in only one of the two is compatible with cell viability. Ideally, if A and B are two synthetic lethal genes, inhibiting B should kill cancer cells with a defect on A, and should have no effects on normal cells. Thus, synthetic lethality can be exploited for highly selective cancer therapies, which need to exploit differences between normal and cancer cells.

**Results:**

In this paper, we present a new method for predicting synthetic lethal (SL) gene pairs. As neighbouring genes in the genome have highly correlated profiles of copy number variations (CNAs), our method clusters proximal genes with a similar CNA profile, then predicts mutually exclusive group pairs, and finally identifies the SL gene pairs within each group pairs. For mutual-exclusion testing we use a graph-based method which takes into account the mutation frequencies of different subjects and genes. We use two different methods for selecting the pair of SL genes; the first is based on the gene essentiality measured in various conditions by means of the “Gene Activity Ranking Profile” GARP score; the second leverages the annotations of gene to biological pathways.

**Conclusions:**

This method is unique among current SL prediction approaches, it reduces false-positive SL predictions compared to previous methods, and it allows establishing explicit collateral lethality relationship of gene pairs within mutually exclusive group pairs.

**Supplementary Information:**

The online version contains supplementary material available at 10.1186/s12859-021-04168-7.

## Background

Most chemotherapeutic agents in use today were discovered by their ability to kill rapidly dividing cancer cells. When administered to patients, these agents also injure rapidly dividing normal cells, thereby causing harmful side effects to patients. For example, doxorubicin, which interferes with the DNA thereby stopping DNA replication in rapidly dividing cancer cells, can also cause congestive heart failure [1]. The severity of such side effects may therefore outweigh the benefits of these therapeutic agents. The key to development of safe and effective anticancer therapies lies in identifying molecular targets and their specific inhibitory compounds in a manner to induce selective lethality, by killing only cancer cells but sparing normal cells.

Cancer cells are genetically different from normal cells. So, highly selective cancer therapies need to exploit the distinctive molecular and cellular traits that sensitize only cancer cells to drugs. One avenue to exploit these genetic differences that has shown considerable promise recently is via synthetic lethality (SL). SL, first defined by Bridges [2] in 1922, refers to the genetic relationship between two (or more) genes where simultaneous genetic defects in both (or all) genes cause cell death but a defect in only one of the genes alone is compatible with cell viability [3, 4].

The concept of SL can be used to choose anticancer drug targets. Specifically, protein products of genes that are synthetic lethal to cancer-causing alterations should theoretically represent excellent targets for anticancer therapies [5, 6]. Ideally, if genes A and B are synthetic lethal, then inhibition of B should kill cancer cells harbouring alterations in A, but should have no effect on normal cells. For example, the inhibition of poly (ADP-ribose) polymerase (PARP) in cancer cells that harbour loss-of-function alterations in breast cancer susceptibility genes BRCA1 and BRCA2 is dramatically lethal to the cells [7, 8]. BRCA-deficient cells show reduced ability to repair DNA double-strand breaks (DSBs) which are lethal forms of DNA breaks. PARP aids restart of stalled replication forks during the DNA replication phase (S) of cell division, converting these to DSBs and promoting their repair by BRCA-mediated (homologous recombination) or alternative DSB-repair (the canonical non-homologous end-joining) pathways. However, inhibition of PARP in cells harbouring BRCA1/2 defects results in accumulation of DSBs beyond a level that is tolerated by these cells, whereas normal cells can still repair their DSBs. While cells with germline knockout of PARP can still survive, lethality is induced by PARP inhibition in BRCA-deficient cells. Indeed the discovery of the BRCA-PARP synthetic lethality has been a pioneering breakthrough, and clinical trials on breast, ovarian, and prostate cancers using PARP-inhibition therapy (olaparib, rucaparib and niraparib) have shown encouraging remission rates in patients while also being well-tolerated (fewer side effects) by patients [9]. Nowadays, SL is considered one of the main engine for anti-cancer drug target discovery [10], further corroborated by recent advances such as CRISPR-based gene editing, which allows to screen a large number of potential drug targets. In particular, systematic tumor sample sequencing have been producing a vast amount of data highly valuable for inferring SL relationships between genes and many computational methods have been proposed to explore them [11].

Mutations in a pair of genes that causes SL are expected to be rarely observed in the same cells. Therefore, we can abductively conclude that a pair of genes is a SL pair when mutations in these two genes are *mutually exclusive*. Assume *P* is a set of patients and *a* and *b* a pair of genes. Let $M_a$, $M_b$ and $M_{ab}$ be the number of patients in *P* harbouring mutations in the gene *a*, in the gene *b* and in both genes, respectively. If *a* and *b* are synthetic lethal we expect the events of a mutation on *a* and a mutation on *b* to be mutually exclusive and $M_{ab}$ to be lower than expected (when the two events are independent). An obvious but naive approach for testing mutual exclusion of mutations in gene pairs is using a hypergeometric test, that assigns to $M_{ab}$ a probability $p_{M_{ab}}$ based on the hypergeometric distribution; one then regards those gene pairs for which $p_{M_{ab}}$ is below an arbitrary threshold to be mutually exclusive.

**Fig. 1:**
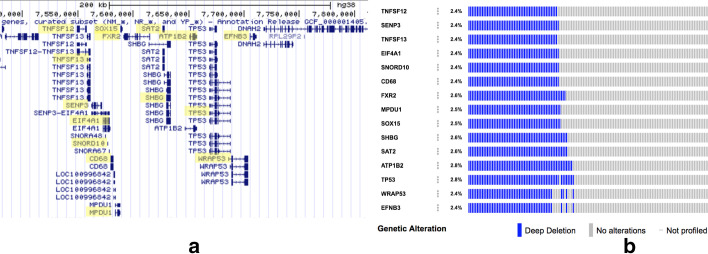
CNA profiles. CNA events usually involve broad regions of the genome. Therefore, close genes generally show very similar CNA profiles. **a** The genomic position of genes close to TP53. **b** the CNA profiles of of the genes highlithed in **a**

However, this approach makes the following assumptions: (1) mutations are mutually independent (i.e., a mutation on a gene *a* does not affect the probability of a mutation of a gene *b* in the same subject), (2) every gene has the same chance to be mutated in a patient, and (3) every patient has the same probability of harbouring a mutation. These assumptions easily do not hold true in the context of human genetics. This is particularly evident if we consider copy number alterations (CNA). In humans, CNAs in genes that are located close by in the human genome (e.g., within 20 cM) tend to be correlated because genetic recombinations take place over large segments of the human genome, sometimes involving the whole arm of a chromosome. Consequently, groups of closeby genes present very similar CNA profiles across patients; e.g., Fig. 1 shows the CNA profiles of genes proximal to TP53. The 15 highlighted genes are located in a focal region of chromosome 17 of just 200 kbp; thus, the deletion of any of them is highly correlated with the deletion of the others, as reflected by their CNA profiles, which are mostly overlapped.

**Table 1:** Classification of the NCI-60 and CCLE cell lines according their mutation state of TP53 and CTCF

	CTCF WT	CTCF MUT
TP53 WT	466	6
TP53 MUT	584	17

Cell lines with TP53 mutated have more than the double probability of having a mutation at CTCF

The presence of certain mutations (e.g. mutations in DNA-damage repair genes) in a patient can cause other mutations to accumulate in that patient. Thus, for a given gene, the probability of being mutated in a patient strongly depends on the patient itself and is not uniform across all the patients in the population. For example, Table 1 reports a classification of 1073 cancer cell lines from the NCI-60 Human Tumor Cell Lines [12] and the Broad Cancer Cell Line Encyclopedia (CCLE) [13] according to their mutations profile on two genes, namely TP53 and CTCF. We observe that 6 out of the 472 cell lines with TP53 wild type present a mutation on CTCF (1.27%), while 17 out of the 601 cell lines with TP53 mutated have a mutation also on CTCF (2.82%). Therefore, the probability of having a mutation on CTCF more than doubles in cell lines harbouring a mutation on TP53, suggesting that the mutations in different genes are actually not independent events. These issues render the hypergeometric distribution inappropriate as a null distribution in the mutual exclusivity test.

Finally, the hypergeometric test considers any pair of genes independently of the other and regardless their position in the genome and the respective neighbour genes. When searching for SL pairs of genes, this approach leads to the generation of many false positives. For example, consider two synthetic lethal genes *x* and *y*. Since the pair *x* and *y* is SL, the two genes’ mutations are also in mutual exclusion. However, any gene *z* in the proximity of *x* is very likely to show a CNA profile similar to the one of *x*; therefore, it is also likely that *y* is in mutual exclusion with any of these genes *z*. Even more, any gene *t* close to *y* is likely to have a CNA profile similar to the one of *y* and therefore to be in mutual exclusion with *x* and with any of the genes *z* close to *x*. Thus, a single SL pair may generate a considerable number of false positives.

The method used in this work mitigates these issues. Genes are clustered into groups according to both their CNA profile and location on the genome. The test of mutual exclusivity is run on pairs of groups, rather than pairs of genes, while the identification of the driver SL gene pair, which induced the mutual exclusivity between the groups, is moved to a separate subsequent phase, where additional information on the genes (e.g., pathway annotations) is considered. To test the significance of the mutual exclusivity, we use a graph-based method, similar to a previous work [14]; in comparison to the hypergeometric test, the graph-based method preserves the different mutation frequencies of different subjects and genes.

## Methods

### Data

We run our pipeline on a set CNA experiments from cBioPortal [15] that comprises the patients of TCGA provisional studies on bladder urothelial carcinoma (BLCA), breast invasive carcinoma (BRCA), colon adenocarcinoma (COADREAD), glioblastoma multiforme (GBM), head and neck squamous cell carcinoma (HNSC), kidney renal clear cell carcinoma (KIRC), brain lower grade glioma (LGG), lung adenocarcinoma (LUAD), lung squamous cell carcinoma (LUSC), ovarian serous cystadenocarcinoma (OV), prostate adenocarcinoma (PRAD) and thyroid carcinoma (THCA). The dataset constructed on the union of these 12 cancer types spans across 6,831 patients and 24,776 genes. Detailed information about the datasets involved in this study is reported in Additional file 1.

The data provided by cBioPortal had been processed by GISTIC2 [16], that, for each patient, assigns a score to each gene. A score of $-2$ indicates that the gene is homozygous deleted while $-1$ indicates a hemizygous deletion and 0 that the gene is wild type. Conversely, positive values of 1 and 2 indicate that the gene is weakly or strongly amplified in the genome. For our analysis we only focused on homozygous deletion and discarded all of the other kinds of alteration. Also, we restricted the analysis to only those genes showing a homozygous deletion in at least 50 patients (approximately 0.8% of the population).

For GARP scores, we used data for a set of 50 breast cell lines, published by Marcotte et al. [17]. For pathway analysis, we used annotations to Reactome pathways [18], as provided by Pathway Commons [19].

### Data representation

We represent the CNA data as a *gene* $\times$ *patient* matrix *M*, as exemplified in Table 2. In *M*, every row corresponds to a gene and every column to a sample/patient. An entry *M*[*i*, *j*] is equal to 1 if the jth patient has a copy number alteration of the ith gene, zero otherwise. The CNA profile of a gene *a* is the row of the matrix *M* that corresponds to the *a* gene and $M_{a}$ is the number of elements equal to 1 in that row.

**Table 2:** Matrix representation of the dataset of CNAs

	Sample 1	Sample 2	$\dots$	Sample M
Gene 1	0	1	$\dots$	0
Gene 2	0	1	$\dots$	1
$\dots$	$\dots$	$\dots$	$\dots$	$\dots$
Gene N	1	0	$\dots$	0

### Gene clustering

We cluster the genes in order to obtain groups of genes that are both close to each other on the genome and show a similar CNA profile across patients. As we do not have any indication on the number of groups, on the cardinality of those clusters and on the maximum distance between the genes in a given group, we use a data-driven procedure. We first group together genes close on the genome and showing similar CNA profile; then, we substitute every group with its *consensus gene* and finally we search for mutual exclusion between pairs of consensus genes.

*Distance* We associate to each pair of genes *g*1 and *g*2 a distance *D* between them, computed as:

$$\begin{aligned} D(g1,g2)={\left\{ \begin{array}{ll} \frac{dist(g1,g2)}{20Mb} + \frac{Pr(g1) \times Pr(g2)}{Pr(g1,g2)}, \quad \textit{if g1 and g2 are on same chr}\\ \infty \quad \textit{otherwise} \end{array}\right. } \end{aligned}$$

where *dist*(*g*1, *g*2) is the distance (in base pairs) between the transcription start sites of the two genes, *Pr*(*g*1) and *Pr*(*g*2) are the empirical probabilities of having a mutation on the gene *g*1 and *g*2 respectively and *Pr*(*g*1, *g*2) is the empirical probability of having a mutation on both the genes. If two genes are located on two different chromosomes their relative distance is set to infinity. Otherwise, their distance depends on both their relative position on the genome and their mutation profiles. Note that the genomic distance between genes appears in the numerator of one of the two components of *D*, hence *D* increases with the genomic distance. Also, the correcting factor of 20 Mbp has been chosen since it corresponds to a distance of 20 cM. For what it concerns the mutations profiles, $Pr(g_1)\times Pr(g_2) < Pr(g_1, g_2)$ when the mutations on the two genes tend to co-occur. Therefore, a strong similarity (overlap) of the two mutations profiles will make the second component of *D* to decrease.

*Clustering Algorithm* As clustering algorithm we select Affinity Propagation [20], where we use as measure of similarity (or affinity) between two genes the inverse of their distance *D*. Compared to other popular clustering algorithms such as *k-means*, Affinity Propagation does not require the number of clusters to be determined before running the algorithm, as it automatically estimates the number of clusters.

*Consensus gene* The output of the Affinity Propagation is a list of clusters (groups) of genes. Affinity Propagation does not constrain the size of the clusters and singletons are allowed. A cluster corresponds to a sub-matrix of *M*, obtained by selecting only a subset of the rows of *M*. Let *C* be a cluster of genes identified by Affinity Propagation and call $M_C$ the sub-matrix of *M* corresponding to *C*. We represent every cluster of genes *C* with its *consensus gene* $c_C$, defined as a vector of length |*P*|, the number of patients, such that every element of $c_C[i]$ is set to 1 if a majority of the genes in *C* are altered in the *i*th patient (i.e., most of the elements in the *i*th column of $M_C$ are equal to 1), and 0 otherwise. The output of this step is a matrix *cluster* $\times$ *patient*, thus having the same number of columns as *M* but fewer rows.

### Calling mutual exclusion between consensus genes

Our method for assessing the mutual exclusivity between two group of genes is composed of two steps: first we compute, for every pair of consensus genes, a score that indicates the “degree” of mutual exclusivity; then, we associate a *p* value to the score so as to understand its significance.

Let *a* and *b* be two consensus genes, and $M_a$, $M_b$ and $M_{a,b}$ the usual counts of mutations and co-mutations. Their score is the *Hamming distance minus intersection* (HDMI), which is defined as the number of patients with exactly one of the two gene mutated minus the number of patients with both genes mutated, normalized over the total number of patients.

$$\begin{aligned} HDMI(a,b) = \frac{M_a + M_b -2M_{ab} - M_{ab}}{|P|} = \frac{M_a + M_b -3M_{ab}}{|P|} \end{aligned}$$

where the component $M_a + M_b -2M_{ab}$ is the *Hamming distance* between binary vectors. Notice that, for fixed values of $M_a$ and $M_b$, higher values of HDMI correspond to more mutually exclusive consensus genes (indeed, HDMI decreases with $M_{ab}$); for an extended overlap between the profiles of *a* and *b* we can obtain negative values of HDMI. Also, HDMI favours consensus genes with many mutations (i.e., to obtain high values of HDMI, high values of $M_a$ and $M_b$ are necessary). This bias is intended, since we believe it to be more useful to favor the genes that are mutated/altered in a larger portion of the population. Due to this bias, it impossible to discern positive and negative cases using a fixed threshold on HDMI; hence we associate the HDMI to a significance value.

**Fig. 2:**
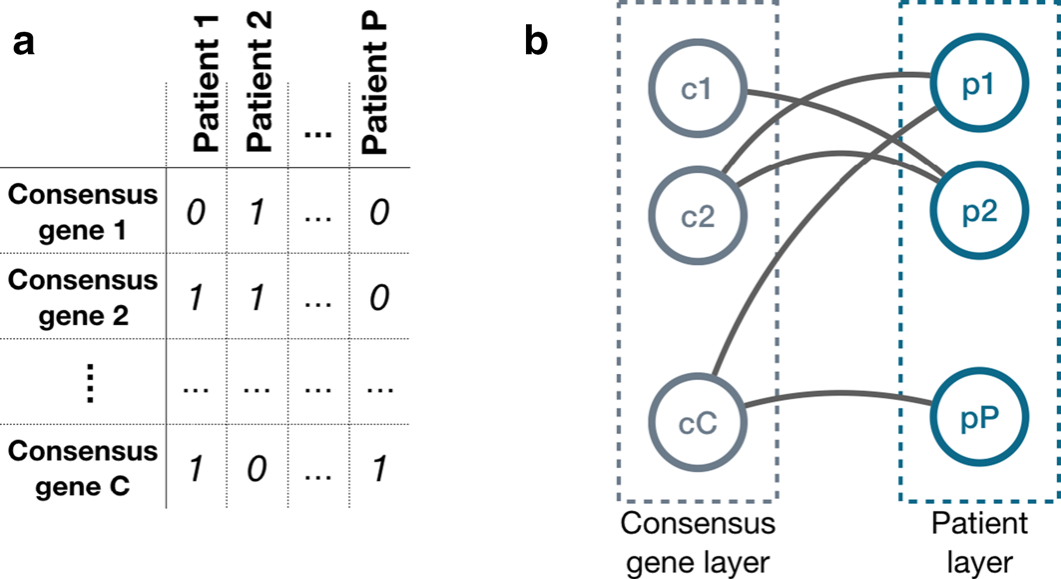
Bipartite graph. The *consensus genes* $\times$ *patients* matrix (**a**) can be interpreted as a contact matrix of a bipartite undirected graph (**b**), in which one layer of nodes corresponds to the consensus genes and the other to the patients. An edge between two nodes is present if and only if the corresponding consensus gene is “mutated” in the corresponding patient

Next we describe the procedure we have designed to associate a significance score to the HDMI of two consensus genes *a* and *b*. First we represent the data in the *consensus genes* $\times$ *patients* binary matrix as a bipartite graph $B=\langle (P \cup C),E \rangle$, where *P* is the set of patients, *C* is the set of consensus genes, and $\{(p,c) \in E: p\in P, c \in C\}$ if gene *c* is altered in patient *p*, as illustrated in Fig. 2.

**Fig. 3:**
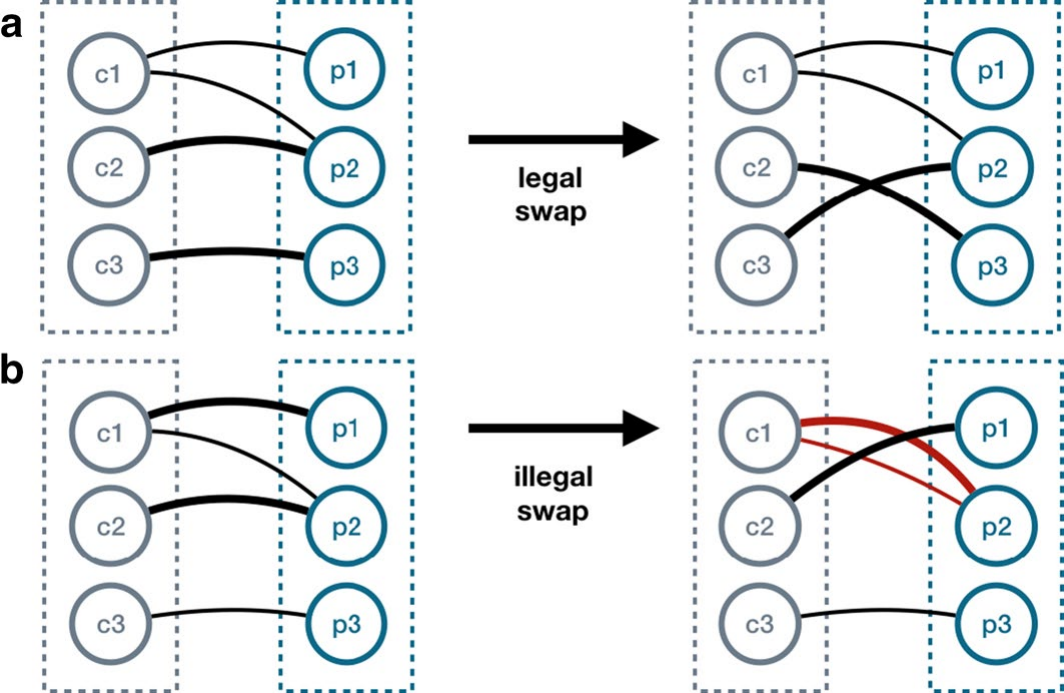
Legal and illegal swaps. A random edge swap may lead to a legal (**a**) situation where no pair of nodes is connected twice and to (**b**) an illegal situation, where two nodes are connected by a pair of edges. In the case of (**b**) the swap is prohibited

Notice that the out-degree of the a consensus gene *a* is $M_a$. If we consider the nodes corresponding to two consensus genes *a* and *b*, we can compute $M_{ab}$ as the number of patient nodes connected to both genes. The method determines if $M_{ab}$ is significant by considering the probability of finding at most $M_{ab}$ co-alteration in a null model. For computing significance, we use a null model made of 10,000 random bipartite graphs, generated from the original one by an edge swapping procedure, consisting of randomly selecting two edges in the graph and swapping their ending points. Pairs of edges ${(p_1,c_1), (p_2,c_2)}$ are randomly picked from the graph such that $(p_1, c_2) \notin E$ and $(p_2 , c _1) \notin E$ and swapped: $E := E \cup \{(p_1,c_2), (p_2, c_1)\} \setminus \{(p_1,c_1), (p_2, c_2)\}$. Doing so changes the pattern of alterations across the patients, but preserves the patient and gene alteration totals; see Fig. 3.

Every random bipartite graph is generated by 100,000 swaps; each of the 10,000 random graphs produced by the procedure has the important property of preserving the degrees of the original graph. Thus, every node of any random graph has exactly the same number of incident edges as the corresponding node in the original graph. This means that the mutation frequencies of consensus genes and patients are preserved.

Given two consensus genes *a* and *b* we are now ready to associate to their HDMI distance a significance. For each random graph *i* we compute the number of patients in which both *a* and *b* are mutated $M^{i}_{ab}$, and we associate to the HDMI the following pvalue:

$$\begin{aligned} p(HDMI(a,b)) = \frac{|\{M^{i}_{ab} : M^{i}_{ab} \le M_{ab}, \quad i= 1,\dots , 10,000 \}|}{10{,}000} \end{aligned}$$

In other words, the *p* value corresponds to the portion of random graphs in which we get a $M^{i}_{ab}$ lower or equal to the observed one. Notice that lower $M^{i}_{ab}$ actually corresponds to higher *HDMI*(*a*, *b*), since the other two components of the measure ($M_a$ and $M_b$) do not vary.

### Identifying synthetic lethality candidates

The previous step produces pairs of gene groups (X, Y) where mutations in the genes in X tend to be mutually exclusive to mutations in the genes in Y. Given that the mutation profiles of the genes within the same group are similar, the HDMI value and the associated *p* value computed on X and Y is a valid approximation of the HDMI and associated *p* value between any pair of genes $x \in X$ and $y \in Y$. Since we postulate that SL implies mutual exclusion, we expect at least one pair of gene $x \in X$ and gene $y \in Y$ to be an SL pair, though we do not know the exact gene pair (*x*,*y*).

We propose two different methodologies to identify the actual SL pairs among the set of candidates. The first one is based on the Gene Activity Rank Profile (GARP) [17] scores for each gene, if these are available. GARP scores measure gene essentiality through siRNA-mediated knock-down screening. For a given cell line, the GARP score is a value (usually in the range $[+5, -10]$) experimentally associated to each gene that measures the essentiality of that gene in that cell line, with lower values indicating higher essentiality. For a pair of mutually exclusive gene groups *X* and *Y*, we leverage GARP scores to find the pair of SL genes by iterating the following procedure for each pair of genes $x \in X$ and $y \in Y$:

- among the set of cell lines for which GARP scores are available, extract 3 sub-sets: (a) cell lines where both gene *x* and gene *y* are wild type, (b) cell lines where *x* is wild type and *y* is mutated and (c) cell lines where *x* is mutated and *y* is wild type;
- compute the median GARP score of gene *x* on the sets (a) and (b);
- compute the median GARP score of gene *y* on the sets (a) and (c);
- call the pair to be SL if the median GARP of *x* on set (b) is below the median GARP of *x* on set (a) and the median GARP of *y* on set (c) is below the median GARP of *y* on set (a).

The relationship in the last point states that *x* becomes more essential in cell lines where *y* is mutated than in cell lines where *y* is wild type and, conversely, *y* becomes more essential in cell lines where *x* is mutated than in cell lines where *x* is wild type. Thus, a mutation on gene *x* compromises the viability of the cell in the cell lines where also *y* is mutated more than in the cell lines where *y* is wild type, and vice versa.

Unfortunately, for some pairs of genes we may not be able to identify the three required sub-sets of cell lines, e.g., in the case where none of the cell lines for which GARP scores are available harbours a mutation of a certain gene.

**Fig. 4:**
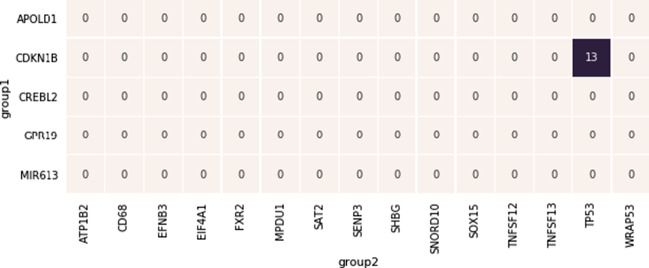
Example of a pair of mutually exclusive groups of genes. Two groups of genes called to be in mutual exclusion are represented respectively on the vertical and horizonal dimension of an heatmap. The numbers in the cells of the heatmap represents the number of common pathways of the two corresponding genes. The *p* value of the count of common pathways between TP53 and CDKN1B is 1.88e−17

For this reason, we introduce a second approach for spotting the correct SL pair of genes. This approach is based on the assumption that two SL genes would likely participate in the same biological pathways. Therefore, we look for those pairs (*x*,*y*) in (*X*,*Y*) that are observed to be in many pathways together, and predict these pairs to be the synthetic lethal pairs. For example, in Fig. 4, we present two groups which were called to be mutually exclusive by our method. As can be seen, among the 75 possible pairs of genes, just one of them, namely the pair made of the CDKN1B and TP53, shares several pathways (e.g., *ErbB Signaling Pathway* [21] and *miRNAs involved in DNA damage response* [22]). The remaining 74 pairs are collateral mutually exclusive. Therefore, their CNA profiles actually show a mutual exclusion, but this is “inherited” from the presumed synthetic lethality of the two near-by genes.

A caveat is that, the pathway-based method is biased in favor of genes that are annotated to many pathways. A possible solution to this problem is to use the hypergeometric test on the sets of pathways associated with the two genes; i.e. test whether the number of pathways shared by the two genes is higher than would be expected when the two genes were independent. This, indeed, is the approach we adopt in the Results Section.

## Results

We present here the results obtained by applying our method to a large dataset of CNA from many patients and compare our predictions with the one provided by the standard procedure based on the hypergeometric test.

### Clustering

**Fig. 5:**
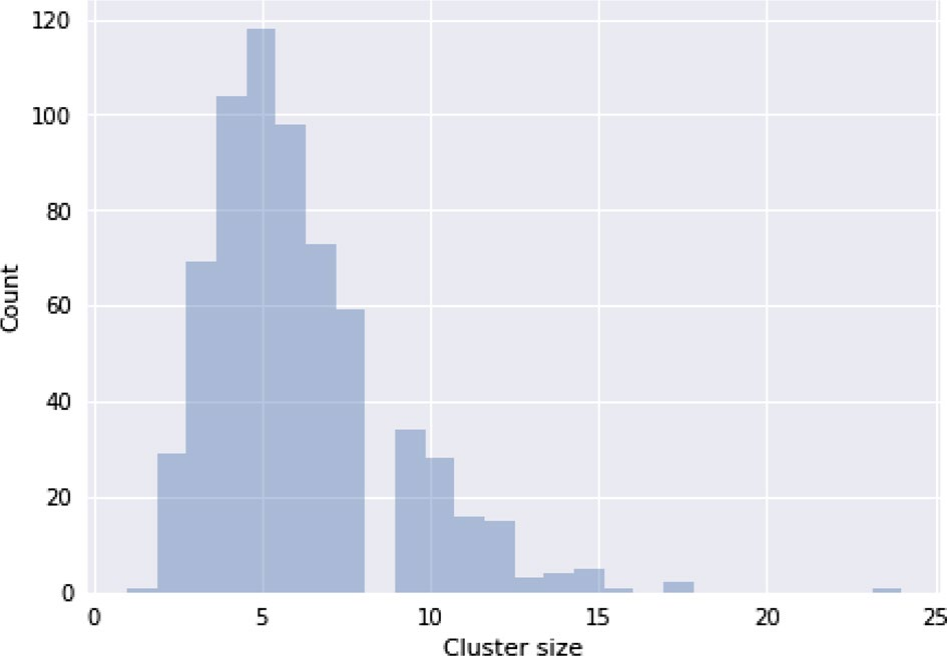
Cluster sizes. Distribution of the sizes of the groups of genes generated by the clustering step

The clustering step on the defined dataset produced 660 groups of genes with their corresponding consensus genes. In Fig. 5 the distribution of the cardinality of the resulting clusters is reported; most groups contain 3–8 genes. Additional file 3 reports the list of genes assigned to each cluster.

### Mutual exclusion for DDR genes

In our test we focused on seven DNA Damage Repair (DDR) genes, namely PTEN, TP53, BRCA2, ATM, CDH1, RB1 and MSH3. Each of these seven genes correspond to a group of genes. Additional file 2 reports the computed pvalue and HDMI score for all the pairs of clusters. For each of the seven groups we identified the set of mutually exclusive partner groups with a *p* value lower than 0.05. Note that each gene has a different threshold value of HDMI that corresponds to a *p* value of 0.05. Then we applied the GARP-based procedure to identify the real SL pair of genes. The results are reported in Table 3. We applied two slightly different variants of the method: In one case, column **garpDD**, for a given gene *g*, we compared cell lines in which *g* is wild type against cell lines in which *g* is homozygous deleted; in the other case, column **garpALT** we compared cell lines in which *g* is wild type against cell lines in which *g* is either homozygous deleted or harbours somatic mutations. The results are reported in the form *n*/*N*, where *N* is the number of pairs for which we have GARP data to perform the test and *n* is the number of cases in which we found at least a pair of genes confirmed by GARP scores.

**Table 3:** Summary of the prediction for 7 DDR genes

Gene	HDMI	Pairs	garpDD	garpALT
PTEN	0.040	71	49/66	54/67
TP53	0.025	51	33/43	40/50
BRCA2	0.030	34	0/00	30/34
ATM	0.020	26	18/24	21/24
CDH1	0.025	33	16/32	18/32
RB1	0.040	52	44/52	45/52
MSH3	0.025	18	8/13	17/18

All the results refer to a *p* value < 0.05. **HDMI** reports the threshold used for the HDMI between the groups; **pairs** is the count of groups found to be in mutual exclusion with the group of the corresponding DDR gene; **garpDD** is the number of pairs for which at least a pair of genes has been validated considering only cell lines with deep deletion, divided by the number of group pairs for which data for the validation are available; **garpALT** as previous, but also considering *somatic mutations*

**Table 4:** Summary of the prediction for 7 DDR genes

Gene	HDMI	Pairs	PW	Validated
PTEN	0.040	71	43	24/35
TP53	0.025	51	32	12/28
BRCA2	0.030	34	15	7/12
ATM	0.020	26	15	6/14
CDH1	0.025	33	15	4/15
RB1	0.040	52	35	16/35
MSH3	0.025	18	11	2/7

All the results refer to a *p* value < 0.05. **HDMI** reports the threshold used for the HDMI between the groups; **pairs** is the count of groups found to be in mutual exclusion with the group of the corresponding DDR gene; **PW** is the number of group pairs for which at least one gene pair passed the hypergeometric test on pathways; **Validated** is the number of group pairs for which at least one of the gene pairs identified by the hypergeometric test on pathways is also confirmed by the GARP test (considering both deep deletion and somatic mutations)

With the first method we were able to check 230 out of 285 pairs of groups (80.7%); for 168 out of those 230 (73%) pairs we found at least one pair of genes confirmed based on GARP scores. With the second method we were able to test 277 pairs (corresponding to the 97.2% of the total). In 225 out of those 277 pairs (81.2%) we found at least a gene pair confirmed based on GARP scores.

### Identification of SL pairs by pathway analysis

We next ran the alternative method based on the comparison of pathway annotations to identify the SL pair of genes, and used GARP scores for validation. For each pair of groups *X* and *Y* predicted to be in mutual exclusion, we are interested in finding at least a pair of genes $x \in X$ and $y \in Y$ such that the hypergeometric test on the pathways annotated to *x* and *y* yields a *p* value lower than 0.05. Then, we validate the predicted SL gene pairs by means of GARP scores.

Results are reported in Table 4: column **pairs** reports the number of mutually exclusive group pairs involving the considered DDR gene; column **PW** reports the number of pairs of groups for which we found at least a pair of genes which passes the hypergeometric test on the pathways; column **Validated** is in the form *n*/*N*, where *N* is the number of cases for which we have GARP data and *n* is the number of validated cases (we regard a pair of groups *X* and *Y* to be validated when at least one of the gene pairs $x \in X$ and $y \in Y$ passing the hypergeometric test on pathways also passes the GARP test).

Notably, for 166 out of 285 (58.2%) pairs of groups we were able to find at least one gene pair passing the hypergeometric test on pathways; for 146 of such pairs we were also able to check the GARP score and in 71 cases (48.6%) at least one of the gene pairs identified by the pathway method were also confirmed through the GARP test.

### Examples of SL predictions

**Fig. 6:**
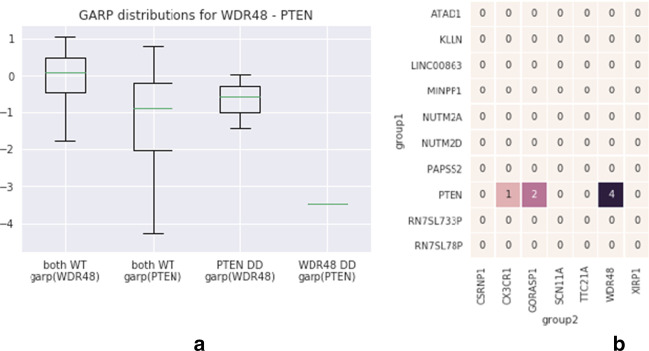
PTEN versus WDR48. **a** The GARP score comparison in different conditions; both PTEN and WDR48 become more essential when the partner is deleted. **b** The matrix of the common pathways between the genes of the two groups. The size of intersection of the set of pathways annotated with PTEN and WDR48 is significant, with Benjamini–Hochberg adjusted *p* value 8.13e−04

**Fig. 7:**
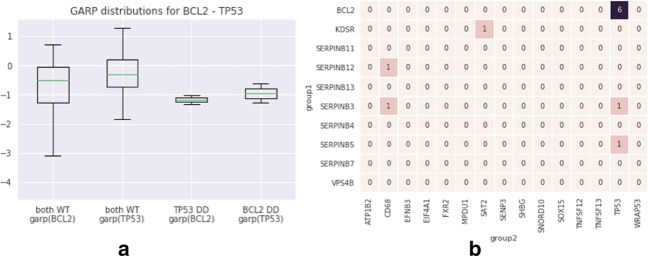
TP53 versus BCL2. **a** The GARP score comparison in different conditions; both TP53 and BCL2 become more essential when the partner is deleted. **b** The matrix of the common pathways between the genes of the two groups. The size of intersection of the set of pathways annotated with TP53 and BCL2 is significant, with Benjamini–Hochberg adjusted *p* value 1.08e−05

Here, we report two interesting examples of SL interactions identified by our method, PTEN-WDR48 (Fig. 6) and TP53-BCL2 (Fig. 7).

In the first example, tumour suppressor PTEN is a phosphatase that is a constituent of the negative feedback loop of the PI3K-AKT pathway, a key serine/threonine signalling pathway responsible for cell growth and proliferation. PTEN controls PI3K-AKT activity by negatively regulating downstream AKT molecules [23]. WDR48 is a serine/threonine phosphatase that regulates human deubiquitinating enzymes (USP 1, 12, 46) to control DNA damage and it is also important in other cellular processes e.g., synaptic transmission, signaling via Akt, Notch and T cell receptor pathways. In this sense, PTEN and WDR48 demonstrate overlapping functions  [24]. Thus, if WDR48 is deleted, the cell may not survive, unless PTEN and other backup genes are active. Conversely, if PTEN (as a tumor suppressor) is deleted, the cell might improve in tumorigenesis; with the inhibition of WDR48 the cell might survive, but it is likely to have functional impairment. This is well reflected by the asymmetry in Fig. 6a: if a cell is WDR48-deficient and we inhibit PTEN, the cell dies. Conversely, if the cell is PTEN-deficient and WDR48 is inhibited, the cell may survive (albeit functional impaired). This may suggest that the use of a PTEN inhibitor in a WDR48-deficient cancer could result in an effective treatment, but not the other way round (i.e., using a WDR48 inhibitor in a PTEN-deficient cancer).

In the second example, both TP53 and BCL2 belong to the programmed cell death or apoptosis pathway. However, while TP53 is pro-apoptotic and triggers cell death upon sensing DNA damage or other triggers during cell cycle, overactivation of BCL2 is anti-apoptotic. Therefore, we expect that in the event of loss of TP53, a simultaneous loss of BCL2 restores the apoptosis of cells [25, 26] (in agreement to Fig. 7a).

### Example of collateral SL pair

The TP53 is the most frequently mutated gene in human cancer; its homozygous deletion often exhibits a co-deletion of the neighbour gene FXR2, which belongs to the Fragile X gene family. In the dataset of CNA that we analyzed in this study the 62% of the patients having a homozygous deletion of TP53 also have FXR2 homozygously deleted.It has been demonstrated that in human cancer it is possible to selectively block cell proliferation by inhibiting, in those cells deleting FXR2, the remaining family member FXR1 [27]. Thus, targeting FXR1 is potentially a therapeutic approach for those human cancers harbouring a homozygous deletion of TP53. We say that TP53 and FXR1 are in a collateral synthetic lethal relationship.

### Comparison with baseline method

To further assess our method, we run the hypergeometric test used in previous work [28] on the same dataset and validated the results using both pathways and GARP scores. Results are reported in Table 5 and show that our method has a higher precision and sensivity (both about doubles) compared to the hypergeometric test, when taking the top *n* predictions by the hypergeometric test (where *n* equals the total number of gene pairs in the mutually exclusive groups identified by our approach).

**Table 5:** Validation of the SL gene pairs identified by means of the hypergeometric test on CNA profiles

Gene	#pairs	hyp-PW	hyp-GARP	our-PW	Our-GARP
PTEN	71	13	19	43	49
TP53	51	14	0	32	33
BRCA2	34	0	4	15	0
ATM	26	3	10	15	18
CDH1	33	5	4	15	16
RB1	52	7	19	35	44
MSH3	18	0	5	22	8

For each DDR gene, we ranked the predictions and considered the top pairs, in the same number as the predictions of our method. The we validated such predictions with both the pathway (**hyp-PW**) and the gene essentiality (**hyp-GARP**) methods. We report the summary of the predictions for 7 DDR genes; all the results refer to a *p* value < 0.05. In order to facilitate the comparison with our method the table also reports the **our-PW** value (which corresponds to the *PW* value of Table 4) and **our-GARP** value (which corresponds to the *garpDD* value of Table 3)

### Example of validation based on survival analysis

**Fig. 8:**
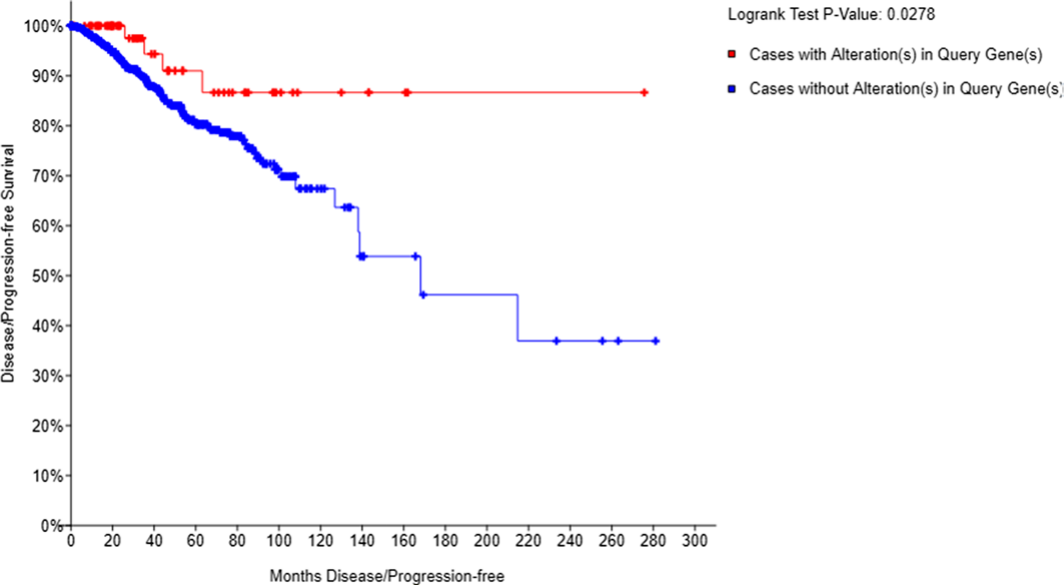
Survival analysis of POLR3D. Homozygous deletion of POLR3D (which is observed in the 8% of breast cancer cases), results in a significantly better disease-free survival

We tested our prediction method on a dataset of $>6800$ CNA experiments involving 24,776 genes. In our experiments, we focused on 7 DDR genes. In total, we identified 660 groups with 12,117 predicted mutually exclusive interactions between the groups. On average, there were slightly over 6 genes per group, with the largest group containing 23 genes, including CDKN2A and CDKN2B respectively. The subnetwork of SL interactions involving DDR genes namely, ATM, BRCA1, BRCA2, CDH1, PTEN, TP53, and RB1, containes 69 groups involving 488 distinct genes. A gene found in several of these groups was POLR3D, a DNA-directed RNA III polymerase subunit, which is lost (hom del, loss of mRNA or protein expression) in about 8% breast cancer cases, however, its loss, in patients for which at least on of the DDR gene is lost , results in significantly better disease-free survival ($p=0.0278$), as shown in Fig. 8. This suggests SL inhibition of POLR3D could be explored as an avenue in DDR-deficient breast cancers.

## Discussion and conclusions

Several computational approaches have been proposed for inferring synthetic lethality pairs of genes from genomic alterations. In 2008 Yeang et al. [29] was the first to publish a method to analyze somatic alteration patterns from large datasets of samples. In 2011 RME [30] used a network analysis method to identify recurrent and mutually exclusive genetic aberrations, while in 2012 Dendrix [31] used a Markov chain Monte Carlo method to address a similar problem. In the same year, Ciriello et al. developed MEMo [14] whose aim is to identify modules of mutually exclusive genes in cancer. MEMo was the first to use the edge swapping approach to test the mutual exclusivity of aberrant events. In 2015, Srihari et al. [28] adopted the hypergeometric test to infer mutually exclusive pairs of genes. More recently, a novel method based on a forward selection algorithm that initially identifies seed pairs of mutually exclusive genes and then expands the selected set [32].

The method we have proposed in this work differentiates from the above for the preliminary step of clustering genes according to their aberration profile across patients and for the approach of testing mutual exclusivity of groups of genes rather than single genes, by means of the novel HDMI measure associated with the edge swapping. Finally, we use additional information (viz. essentiality data and pathway annotations) to identify driver gene pairs.

In our approach, we explicitly took care of confounding due to genetic linkage (i.e., neighbouring genes in the genome have highly correlated profiles of CNA); thus we first cluster proximal genes whose CNA profiles are similar, then predict mutually exclusive group pairs on the basis of mutation profiles, and finally identify the SL gene pairs within each group pairs. We proposed two different methods for selecting the pair of SL genes; the first is based on the gene essentiality measured in various conditions by means of GARP score, while the latter leverages the annotations of gene to biological pathways. This aspect is unique among current SL prediction approaches; it has the effect of reducing false-positive SL predictions, as well as making explicit collateral lethality relationship of other gene pairs within mutually exclusive group pairs.

We estimate from our result (Table 3) that 73–81% of the mutually exclusive group pairs are valid (i.e., they each contains at least one SL gene pair). Hence at the level of group pairs, we have predicted 285 mutually exclusive groups at 73–81% precision. For what concerns the identification of the SL gene pair, we estimated from our result (Table 4) that 49% of the predicted SL gene pairs are valid (i.e., the pair selected by the pathway-based method is also confirmed by GARP scores), thus the precision of our method is 49% and the sensitivity is 32% (= 71/225).

Beside predicting SL pairs, our method is also able to identify collateral lethal pairs, by considering other gene pairs in mutually exclusive group pairs. Recall that when a gene *x* is often deleted along with a gene *y* and a gene *z* is in a synthetic lethality relationship with *x*, then *z* is often also in a collateral lethality relationship with *y*. That is, inhibiting *z* in cancer cells bearing y deletion is often lethal to these cells. Thus our mutually exclusive group pairs serve as an expanded list of drug targets for cancers bearing any of the 7 DDRs studied here.

## Supplementary Information

**Additional file 1.** The list of the datasets involved in this study.

**Additional file 2.** The p value and the HDMI score for each pair of gene clusters.

**Additional file 3.** 660 gene clusters identified.

## Data Availability

The datasets of CNA analysed during the current study are available in the cBioPortal repository, https://www.cbioportal.org/datasets. The dataset of pathway annotations analysed during the current study is available in the Pathway Commons repository, https://www.pathwaycommons.org/archives/PC2/v11/PathwayCommons11.reactome.hgnc.gmt.gz. The dataset of gene essentiality GARP score analysed during the study is available from the corresponding author on reasonable request.
